# Light Energy Dose and Photosensitizer Concentration Are Determinants of Effective Photo-Killing against Caries-Related Biofilms

**DOI:** 10.3390/ijms21207612

**Published:** 2020-10-15

**Authors:** Abdulrahman A. Balhaddad, Mohammed S. AlQranei, Maria S. Ibrahim, Michael D. Weir, Frederico C. Martinho, Hockin H. K. Xu, Mary Anne S. Melo

**Affiliations:** 1 Ph.D. Program in Dental Biomedical Sciences, University of Maryland School of Dentistry, Baltimore, MD 21201, USA; aabalhaddad@umaryland.edu (A.A.B.); Malqranei@umaryland.edu (M.S.A.); mariasyibrahim2@gmail.com (M.S.I.); MWeir@umaryland.edu (M.D.W.); fmartinho@umaryland.edu (F.C.M.); 2 Department of Restorative Dental Sciences, College of Dentistry, Imam Abdulrahman Bin Faisal University, Dammam 34212, Saudi Arabia; 3 Department of Preventive Dental Sciences, College of Dentistry, Imam Abdulrahman Bin Faisal University, Dammam 34212, Saudi Arabia; 4 Department of Advanced Oral Sciences and Therapeutics, University of Maryland School of Dentistry, Baltimore, MD 21201, USA; 5 Division of Operative Dentistry, Department of General Dentistry, University of Maryland School of Dentistry, Baltimore, MD 21201, USA

**Keywords:** dental caries, low-level light therapy, photochemotherapy, photodynamic, reactive oxygen species, *Streptococcus mutans*

## Abstract

Caries-related biofilms and associated complications are significant threats in dentistry, especially when biofilms grow over dental restorations. The inhibition of cariogenic biofilm associated with the onset of carious lesions is crucial for preventing disease recurrence after treatment. This in vitro study defined optimized parameters for using a photosensitizer, toluidine blue O (TBO), activated via a red light-emitting diode (LED)-based wireless device to control the growth of cariogenic biofilms. The effect of TBO concentrations (50, 100, 150, and 200 μg/mL) exposed to light or incubated in the dark was investigated in successive cytotoxicity assays. Then, a mature *Streptococcus mutans* biofilm model under sucrose challenge was treated with different TBO concentrations (50, 100, and 150 μg/mL), different light energy doses (36, 108, and 180 J/cm^2^), and different incubation times before irradiation (1, 3, and 5 min). The untreated biofilm, irradiation with no TBO, and TBO incubation with no activation represented the controls. After treatments, biofilms were analyzed via *S. mutans* colony-forming units (CFUs) and live/dead assay. The percentage of cell viability was within the normal range compared to the control when 50 and 100 μg/mL of TBO were used. Increasing the TBO concentration and energy dose was associated with biofilm inhibition (*p* < 0.001), while increasing incubation time did not contribute to bacterial elimination (*p* > 0.05). Irradiating the *S. mutans* biofilm via 100 μg/mL of TBO and ≈180 J/cm^2^ energy dose resulted in ≈3-log reduction and a higher amount of dead/compromised *S. mutans* colonies in live/dead assay compared to the control (*p* < 0.001). The light energy dose and TBO concentration optimized the bacterial elimination of *S. mutans* biofilms. These results provide a perspective on the determining parameters for highly effective photo-killing of caries-related biofilms and display the limitations imposed by the toxicity of the antibacterial photodynamic therapy’s chemical components. Future studies should support investigations on new approaches to improve or overcome the constraints of opportunities offered by photodynamic inactivation of caries-related biofilms.

## 1. Introduction

Carious lesions on restored teeth recur at alarming rates. The high prevalence of failed restorations has made secondary caries a pernicious problem [1]. The onset of primary or recurrent carious lesions is triggered by the biofilm growth over the tooth surface or tooth–material interface [2,3]. Caries-related biofilms are composed of a densely filled community of microbial cells. These bacterial cells can produce and survive in acidic environments and surround themselves with an exopolysaccharide (EPS)-rich matrix [4,5]. Although this biofilm is composed of many different microbial species, the leading role in its formation and pathogenicity is attributed to *Streptococcus mutans* [6,7].

**Figure 1:**
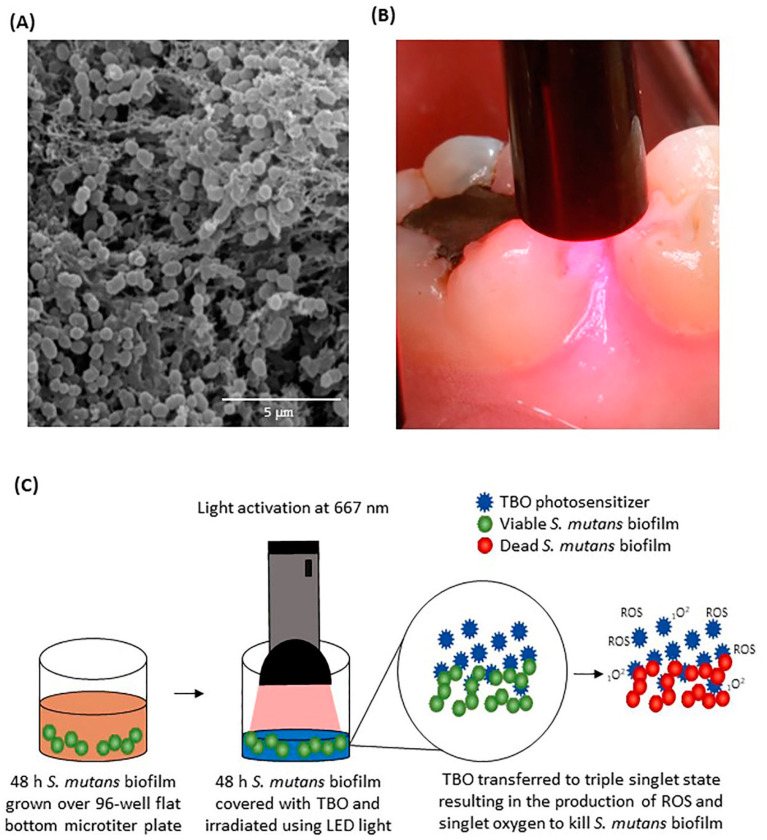
(**A**) Scanning electron microscopy image illustrating the 3D complex structure of *S. mutans* biofilms; (**B**) intra-oral photograph illustrating the clinical application of antibacterial photodynamic therapy (aPDT) to target biofilm growth at the proximal surface of the permanent first molar; (**C**) a schematic drawing showing the methodological design of the study. *S. mutans* biofilm was grown for 48 h, and then irradiated with different concentrations, different energy doses, and different incubation time using LED light source and TBO as a photosensitizer.

In this context, *S. mutans* presents a remarkable ability to survive changes in pH and oxygen tension and produce EPS matrix synthesis to firmly attach to substrates (Figure 1A) [8]. As dental caries is a common biofilm-dependent oral disease, in the presence of sucrose, *S. mutans* biofilms can promote its progression [9], especially in areas inside the oral cavity where the mechanical removal of biofilms by brushing is difficult [10]. Besides, dental composite restorations, composed of resin monomers, may present leachable compounds that stimulate bacterial growth [11]. Therefore, anti-biofilm strategies that can effectively minimize and modulate cariogenic biofilms are currently in demand [12].

Antimicrobial photodynamic therapy (aPDT), also known as photoactivated disinfection, provides a promising approach to inactivate pathogens for biofilm control in dentistry, as illustrated in Figure 1B. aPDT has shown encouraging results against several oral microorganisms without inducing bacterial resistance [13]. The use of aPDT to combat oral biofilms has been a promising approach, where this strategy targets cariogenic biofilms and could help prevent the onset and progression of dental caries [14,15]. In this approach, photosensitizers, followed by light irradiation at a specific wavelength, offer a noninvasive method to target pathogenic biofilms in many dental sites [16]. The mechanism behind aPDT is based on prompting oxidative photo-damage of the targeted bacteria. This process is achieved via two different mechanisms, type I and II [17,18]. In type I, exciting the photosensitizer to the triplet state allows the photosensitizer to interact with the surrounding molecules via electron or hydrogen exchange to generate reactive oxygen species (ROS.). In type II, the interaction occurs between the excited photosensitizer and oxygen molecules in or around the cells resulting in singlet oxygen (_1_O^2^) production. Both approaches can happen at the same time to exert diverse antibacterial activities [18,19,20].

From all photosensitizers that absorb light energy and catalyze the formation of ROS, for aPDT, toluidine blue O (TBO)-mediated aPDT has demonstrated biofilm-eradication or a substantial reduction of cariogenic species [21]. TBO can penetrate easily through the bacterial membrane as it has a transmembrane permeability coefficient higher than other photosensitizing solutions, a fact that possibly makes TBO more effective in bacterial destruction [22]. On the other hand, the amount of ROS generated by a photoactivated photosensitizer is correlated to the absorbed light energy. Therefore, applying the appropriate light parameters such as wavelength and energy dose (irradiation time × power) can affect the dosimetry of aPDT [23]. 

The extent to which aPTD dosimetry influences the bacterial reduction of caries-related biofilm is observed in conflicting outcomes in the literature [24]. Some studies display robust bacterial reduction for *S. mutans* and *Lactobacillus casei* growth in vitro [25], in situ [26], and in vivo [14,15], while others show limited effectiveness against cariogenic biofilms [27,28]. These contradictory outcomes are mainly due to the use of different parameters related to the concentration of TBO, the dose of light energy, and the incubation time before irradiation.

Determining the optimal parameters to target dental biofilms will ensure that the targeted areas inside the mouth are given appropriate doses to kill pathogens, limit over-treatment, and prevent side-effects. From an intent-to-treat perspective, dosimetry is undoubtedly a critical issue for high efficacy and patient safety in aPDT [16,18]. The lack of optimal parameters can opposite the use of aPDT in dental biofilm control as the treatment is associated with unpredictable response rates or a failure to archive clinically acceptable parameters.

Accordingly, this study aims to evaluate the most effective, feasible, and biocompatible concentration, light energy dose, and incubation time to target a 48 h *S. mutans* biofilm using TBO and a narrow-band red light-emitting diode for aPDT.

## 2. Results

### 2.1. Spectra of TBO and LED

**Figure 2:**
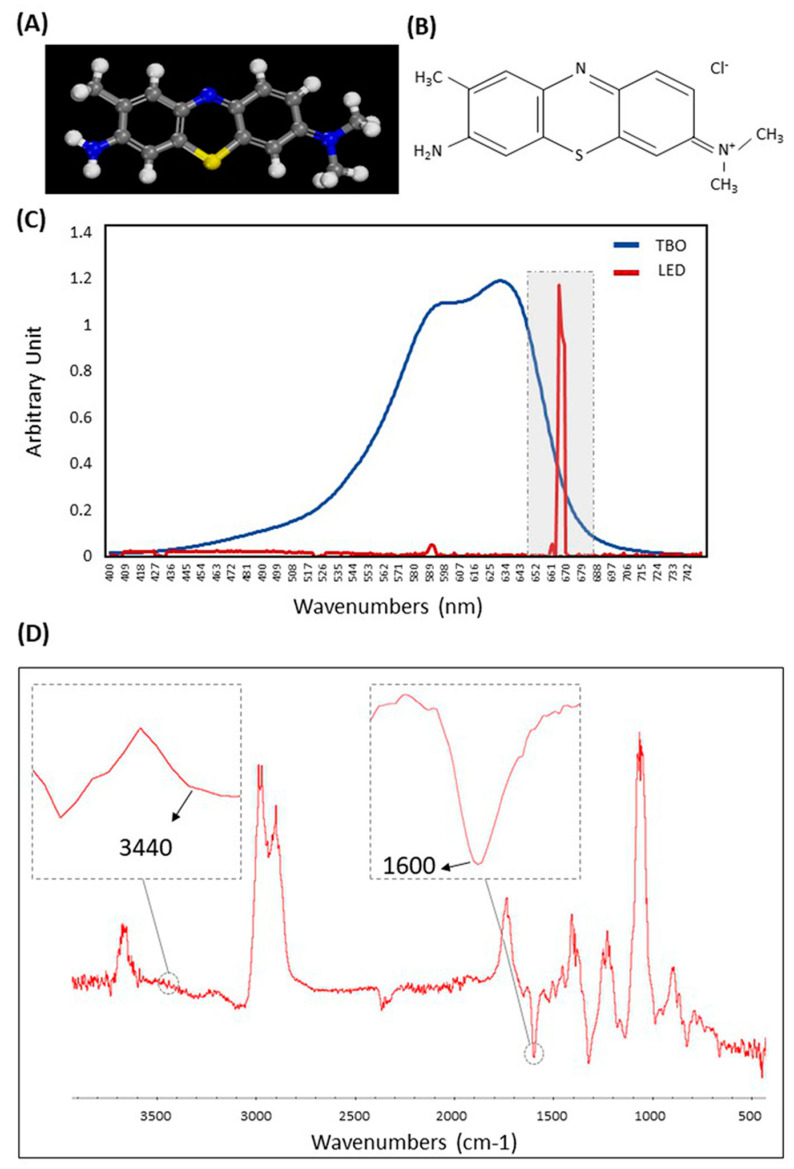
(**A**,**B**) The skeletal structure and the 3D chemical molecular structure of TBO. (**C**) The absorption spectrum of TBO and the maximum peaks absorption at 594 and 632 nm overlapping with the LED spectrum. (**D**) Fingerprint regions of ATR-FTIR spectrum for TBO.

Figure 2A,B displays the skeletal structure and the 3D chemical molecular structure of TBO. The skeletal chemical structure presents sulfur and secondary amine in the middle, primary amine, and methyl group at one quaternary amine at the opposing side. In Figure 2C, the TBO absorption spectrum and the LED emission spectrum are displayed. The TBO presents an absorption band between 550 and 650 with two different peaks at 594 and 632 nm. The emission spectrum peak of the LED source was 667 ± 3 nm, which was overlapped with the peak absorbance of TBO. Figure 2D illustrates the FTIR spectrum of the TBO absorbance. The aromatic ring’s band of the TBO (≈1600 cm^−1^) can be observed. Additionally, the N-H stretch band due to the primary amine (≈3340 cm^−1^) is shown.

### 2.2. TBO Cytotoxicity

**Figure 3:**
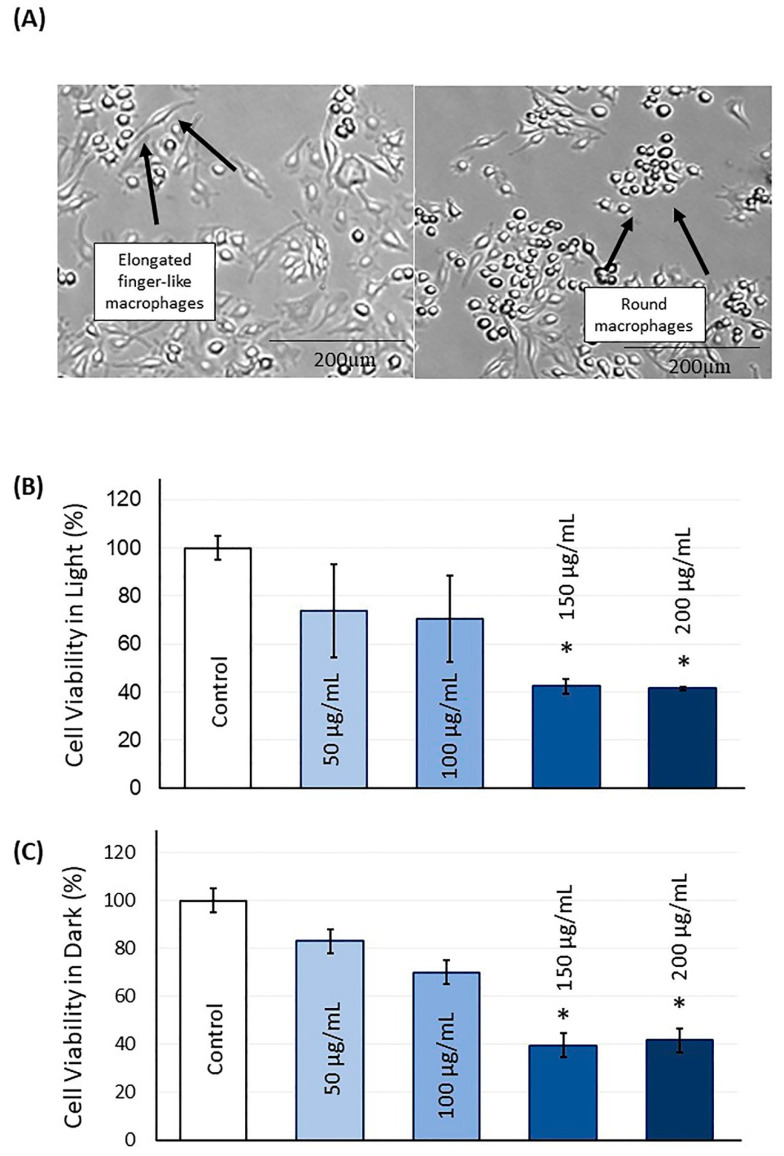
(**A**) Illustrative images displaying the macrophages’ differentiation into elongated finger-like and round cells are the normal differentiation of this cell line. The cytotoxicity of different concentrations of TBO exposed to light (**B**) and in the dark (**C**). Values indicated by different letters are statistically different from each other (* *p* < 0.05).

Figure 3 shows the cytotoxicity of the TBO photosensitizer against the macrophage cell line (*n* = 4). Using TBO concentrations of 50 and 100 μg/mL, either in light or dark, was associated with viability higher than 70%. Using TBO concentrations of 150 and 200 μg/mL resulted in a significant cytotoxicity compared to the control (*p* < 0.05; power of analysis = 100%).

### 2.3. TBO Determining Parameters and S. mutans Photoinactivation

**Figure 4:**
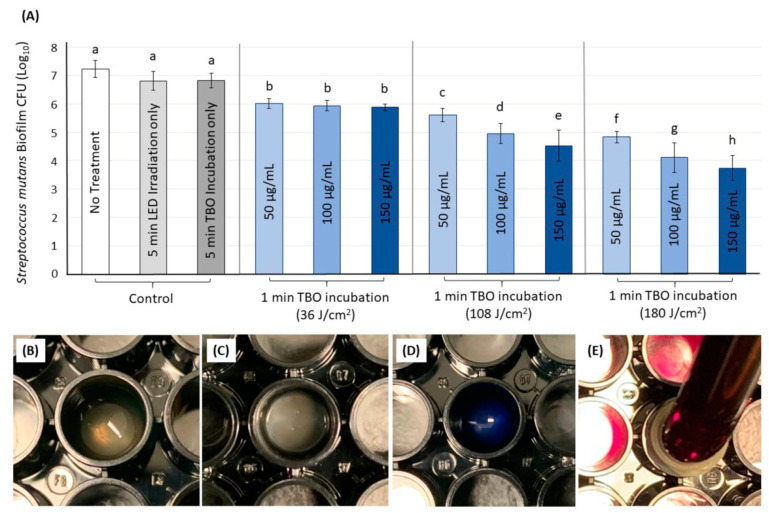
(**A**) The effect of different TBO concentrations and LED light energy doses on *S. mutans* biofilm. Values indicated by different letters are statistically different from each other (*p* < 0.05). The design of the experiment is illustrated in (**B**–**E**). The biofilm was grown for 48 h (**B**). The media was aspirated (**C**). Then, TBO was applied (**D**) and irradiated via the LED light source (**E**).

Figure 4A displays the log reduction (mean and standard) of *S. mutans* biofilm (*n* = 9) achieved by each treatment considering the TBO concentration and energy density. The log values ranged from 7.24 to 3.74, with the highest value for the biofilms treated with 150 µL and 180 J/cm^2^ and the lowest for the control group with no treatment.

The two-way ANOVA analysis showed that both TBO concentration and energy density dose were significantly determinant factors in increasing the effectiveness of aPDT (*p* < 0.001). A significant interaction was observed between the TBO concentration and energy density dose concerning the aPDT effect (*p* < 0.001). No significant difference was found between wells with no treatment (7.24 ± 0.30) and wells treated with light irradiation for 5 min or TBO incubation for 5 min without irradiation (*p* > 0.05; power of analysis = 100%).

The inhibition of 1–3.5-log was observed compared to the control groups in a dose-dependent manner. In comparison to control, the log reduction on *S. mutans* biofilm at the energy density of 36 J/cm^2^ was approximately 1-log (*p* < 0.05), regardless of the used concentrations. When the energy density was increased up to 108 J/cm^2^, significant inhibition of 1.5–2.5-log was observed compared to the control groups in a dose-dependent manner, as increasing the concentration was associated with more inhibition (*p* < 0.05). Three and 3.5-log_10_ reductions were achieved when 100 and 150 μg/mL of TBO, respectively, were activated with 180 J/cm^2^ energy density compared to the control (*p* < 0.05; power of analysis = 100%).

**Figure 5:**
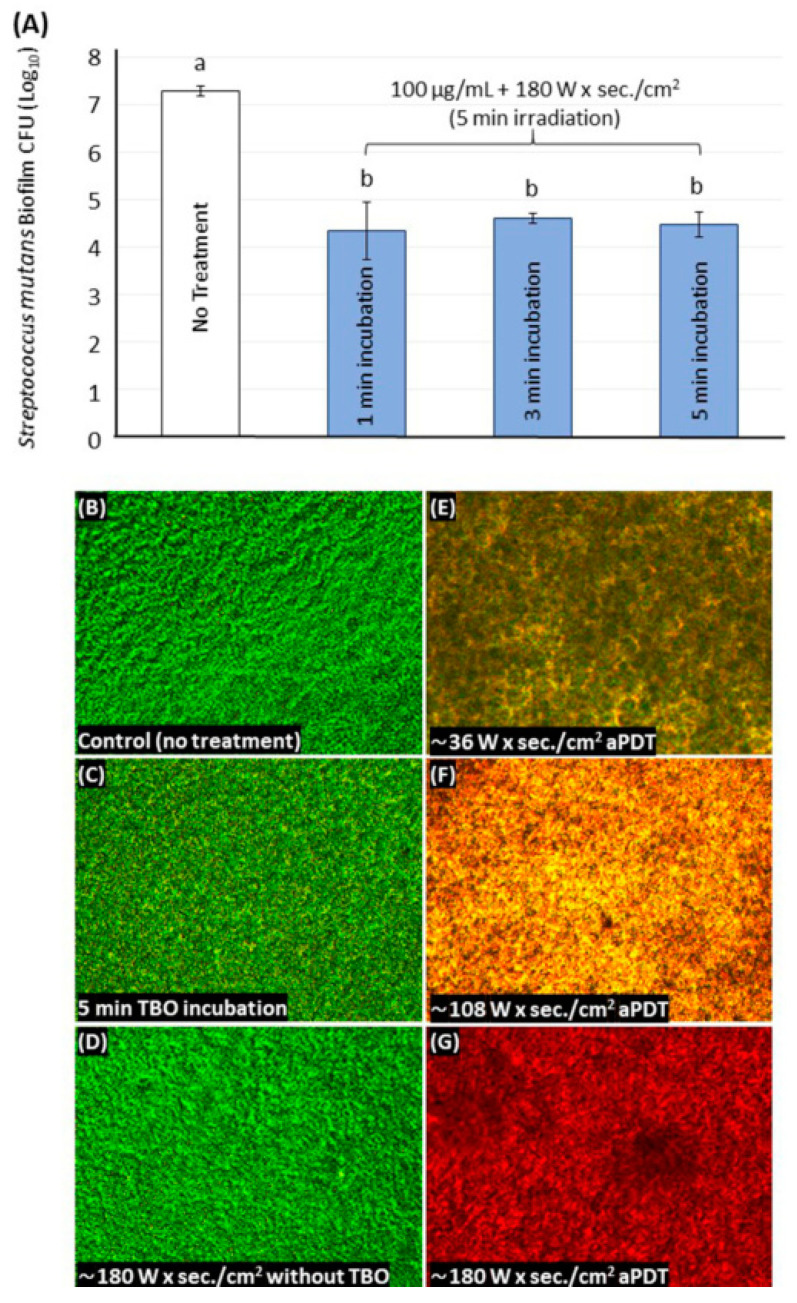
The effect of different incubation times on *S. mutans* biofilm using 100 μg/mL of TBO (**A**). Values indicated by different letters are statistically different from each other (*p* < 0.05). Live/dead images for the *S. mutans* biofilm following no treatment (**B**), 5 min of TBO incubation with no light activation (**C**), light activation only without TBO (**D**), and aPDT treatment with different light energy doses using 100 μg/mL of TBO (**E**–**G**).

Figure 5A demonstrates the effect of increasing the TBO incubation time before irradiation on the *S. mutans* biofilm inhibition (*n* = 9). The energy density of 180 J/cm^2^ was used with 100 μg/mL of TBO. Increasing the incubation time from 1 to 3 or 5 min did not result in an increased antibacterial effect (*p* > 0.05).

### 2.4. Live/Dead Assays for the S. mutans Biofilm 

Figure 5B–G displays the live/dead images for different wells (*n* = 3). Untreated wells (control) and wells were treated with only LED or TBO with no aPDT were associated with a considerable amount of viable *S. mutans* colonies indicated by the green color (Figure 5B–D). However, following the aPDT treatment using different energy density doses with 100 μg/mL of TBO, dead, and compromised colonies indicated in the red color were observed (Figure 5E–G). More dead colonies were seen as the energy density dose was increased from 36 to 180 J/cm^2^.

## 3. Discussion

Antibacterial photodynamic is an attractive anti-biofilm strategy against cariogenic biofilms [29]. However, the application of aPDT for biofilm control is highly dependent on dosimetric parameters. Light energy (fluence), photosensitizer concentration, and incubation time interactions were investigated as determinants to predict treatment effectiveness.

In our study, TBO was selected as a photosensitizer due to its in vitro efficiency, low toxicity to human cells, high rate of ROS generation, and excellent versatility regarding its large band of absorption, which allowed activation by many light sources [30]. TBO belongs to the non-porphyrin, phenothiazinium photosensitizers family [31]. This family of photosensitizers can selectively aim and accumulate inside the mitochondria, compromising the targeted cells [31].

To ensure the TBO biocompatibility with maximum bacterial reduction, different TBO concentrations were investigated. The most commonly reported TBO concentration in the literature to convey high aPDT efficiency against dental biofilms is 100 µg/mL [15,25,32,33]. Therefore, a range of TBO concentrations from 50 to 200 µg/mL were investigated. Additionally, from a clinical perspective, high TBO concentrations may adversely stain the surrounding tissues during aPDT application, especially in the anterior area where the esthetic is important. We report dark toxicity of TBO at 150 and 200 µg/mL, concentrations that also induce similar light toxicity. A high DNA fragmentation after aPDT with TBO at high concentration pointed out that apoptosis is the preferential mode of cell death involved under these conditions [34]. The imperative cytotoxic effect of TBO up to 100 µg/mL with a decrease of less than 40% in optical density suggests that an even lower concentration could be used for aPDT.

As a mandatory step for aPDT, the irradiation of photosensitizers (PS) is considered one of the main determinants for a robust bacterial reduction outcome. Here, we used an LED light source and not a laser to investigate the effect of different energy doses. Most studies investigating the antibacterial effect of TBO-mediated aPDT against cariogenic pathogen employed LED as it produces a low power output, which is beneficial to not causing damage to adjacent tissues, and reduced cost compared to red lasers [25,32]. Another factor favoring LED use is its classification as non-coherent light presetting an amplitude in emitted light waves [35]. The LED chosen for this study presents a bandgap of 8 nm that overlaps the absorption band of TBO. The selected range of energy from 36, 108, and 180 J/cm^2^ was also calculated considering the LED parameters and irradiation periods congruent with several previous reports in the literature.

Our series of investigations on the range of different energy density doses found higher *S. mutans* biofilm inhibition associated with increasing the energy dose. With aPDT at 36 J/cm^2^ (corresponding to 1 min irradiation time), the treatment has delivered a significant log_10_ reduction in relation to control; but the increase of TBO concentration was not relevant. The lack of enhancement of the bacterial reduction outcomes may be attributed to insufficient energy to excite the cells’ photosensitizer uptake even at the highest concentration.

On the other hand, when the energy dose is triplicate and delivered at 108 J/cm^2^, the TBO concentration started to play a critical role in the bacterial reduction. Increasing the TBO concentration was relevant to reach a greater log_10_ reduction. With aPDT applying 180 J/cm^2^, bacterial reduction follows a similar trend to that observed with 108 J/cm^2^. The TBO concentrations of 100 and 150 µg/mL combined with 180 J/cm^2^ delivered in 5 min of irradiation promoted 3 and 3.5-log_10_ bacterial reduction, respectively. Herein, the irradiation time was not increased to more than 5 min as it may carry physical hazards to the operator [36] such as generating non-ionizing radiation by the LED, increasing the risk of heating the dental pulp [37], and imparting discomfort to the patient with a long period of operation time.

Another parameter frequently reported with high variability is the incubation time [38]. The incubation time represents the period where the TBO remains in contact with the *S. mutans* biofilm before irradiation [38]. During this period, the TBO may bind to the plasma membrane and/or penetrate the bacterial cells. Here, we investigated the three most commonly reported periods for TBO incubation, 1, 3, and 5 min [21], associated with our most efficient and biocompatible parameters (180 J/cm^2^; TBO at 100 µg/mL). It was shown in this study that increasing the incubation time had no direct effect on aPDT outcomes. Likewise, previous reports have shown no expressive effect of the incubation time on the bacterial reduction of cariogenic biofilms [39,40]. Although we aim to shorten the incubation time for practicality, further investigations are needed to explore other approaches to enhance the TBO penetration through the cariogenic biofilm.

As limitations, while we can attest to the robust effect of aPDT on cariogenic pathogens in vitro, some drawbacks encountered in clinical situations may compromise the effect in vivo. For instance, well-described environmental stress factors present in vivo conditions activate the biofilm mode of growth, such as frequency and amount of sugar intake, sublethal doses of antimicrobials, nutrient shortage, and inflammatory response or impaired availability of O_2_ on some deep layers of mature biofilms [41,42]. As a result, agents demonstrating suitable antibacterial activities against monospecies biofilm may face difficulties to exert similar action against multispecies biofilms. Moreover, the propagation of light to the infection site could be hampered by local factors such as interproximal spaces, where the tooth–material interface is prone to biofilm accumulation, and the potential inactivation of ROS by saliva and gingival crevicular fluid should be taken into account [41,42].

The reported outcomes are not surprising but critical for TBO-mediated aPDT targeting caries-related biofilms. In most studies, the determining parameters are not reported or only performed to support other assays. Investigators, then, feel the need to start a set of experiments to define a start point for their experiments. By utilizing determining parameters, the aPDT performance can be further investigated in future studies, and other influencing factors can also be considered.

Finally, in light of our observations, we suggest that the dosimetry is based on incubation time of 1 min; TBO concentration at 100 µg/mL; energy dose of 180 J/cm^2^ delivered over 5 min irradiation might be effective and safe for photo-killing of caries-related biofilms. Future studies should support investigations on new approaches to improve or overcome the constraints of opportunities offered by photodynamic inactivation of caries-related biofilms when the maximum bacterial reduction is not reached.

## 4. Material and Methods

### 4.1. Photosensitizer TBO and Light-Emitting Diode (LED)

The photosensitizer TBO (#T3260, Sigma-Aldrich, St. Louis, MO, USA) was dissolved in deionized water, filtered, and stored in the dark. TBO has the following chemical formula; (C_6_H_4_(CH_3_)NH_2_)_2_ (the C.A.S. no. 92-31-9) as it is illustrated in Figure 2A,B. It presents a molar mass of 305.83 g/mol. The TBO absorption peaks (594 and 632 nm) were assessed via ultraviolet-visible optical absorption spectrometry (SpectraMax M5, Molecular Devices, Sunnyvale, CA, USA). The TBO spectrum also was captured by Fourier-transform infrared spectroscopy (FT-IR) (Nicolet 6700, Thermo Fisher Scientific, Waltham, MA, USA).

The light source selected in this study was a light-emitting diode (LED; photo-activated disinfection (PADLight-F3WW, Beijing, China). This light source has a narrow spectrum emission ranging from 664 to 670 nm and a predominant wavelength at 667 nm. The LED light has a cylindrical tip with a diameter of 6.0 mm to distribute the light. Irradiation was performed in a non-contact mode with a diffused beam at 2.0 mm working distance. A power meter Lasermate (Coherent Inc., Santa Clara, CA, USA) was used to measure the peak power. The maximum output power of 180 mW at 2 mm distance was determined. The red LED’s fluorescence emission spectrum was investigated using a light spectrometer (Thorlabs Inc., Newton, NJ, USA).

The surface area of the LED tip was calculated as 0.302 cm^2^ $\left(A=\pi r^{2}\right)$. 

Three energy density doses (36, 108, and 180 J/cm^2^ with a respective irradiation time of 1, 3, and 5 min) were calculated based on the following equation:

$$Energy\;density\;\left(\frac{J}{{\mathrm{cm}}^{2}}\right)=Fluency=\frac{power\;density\;\left(W\right)\times time\;\left(\sec\right)}{A\;\left({\mathrm{cm}}^{2}\right)}$$

where power density is:

$$Power\;density=\frac{light\;intensity\;\left(W\right)}{A\;\left({\mathrm{cm}}^{2}\right)}$$

### 4.2. Light and Dark Cell Cytotoxicity Assay

The cytotoxicity of TBO was examined via MTT colorimetric assay, as previously described [43], with minor modification. At a density of 10,000 cells/well, RAW 264.7 mouse monocyte-macrophage cells (ATCC^®^ TIB-71^TM^) were seeded in a 24-well culture plate containing Dulbecco’s Modified Eagle Medium (DMEM; Thermo Fisher Scientific, Inc., Waltham, MA, USA) and supplemented with 10% fetal bovine serum (FBS; Thermo Fisher Scientific, Inc., Waltham, MA, USA), 100 μg/mL penicillin, and 100 μg/mL streptomycin.

On the following day, different concentrations of TBO (50, 100, 150, 200 μg/mL dissolved in sterile water) were added, and the wells were either kept in the dark (dark cytotoxicity) or exposed to indoor room light (light cytotoxicity) for 48 h (*n* = 4). Then, the old stained cell medium was aspirated, the cells were washed with phosphate-buffered saline (PBS), and a new fresh medium was added. The number of viable cells was quantified using MTT colorimetric assay by the cleavage of tetrazolium salts. 3-(4-5-dimethlthiazol-2-yl) 2-5-diphenyl tetrazolium bromide (MTT) salt was added to each well and incubated for 3 h at 37 °C to form the blue formazan. The chemical reaction was stopped by adding MTT solubilization solution, and the absorbance was measured at 570 nm via a microplate reader (SpectraMax M5, Molecular Devices, Sunnyvale, CA, USA).

### 4.3. S. mutans Biofilm Model

*S. mutans* UA159 obtained from the American Type Culture Collection (A.T.C.C., Manassas, VA, USA) was cultured overnight in brain heart infusion (BHI) broth (Sigma-Aldrich, St. Louis, MO, USA.) at 37 °C and 5% CO_2_ incubator. The *S. mutans* culture was adjusted to 1 × 10^8^ colony-forming units (CFU)/mL (_OD600_ = 0.9) [44] and diluted 1:20 with a fresh BHI broth supplemented with 2% sucrose. Each well of a black 96-well plate with a clear bottom received 200 μL of the inoculum and was incubated for 24 h at 37 °C in a 5 % CO_2_ incubator. Media was changed after 24 h, and 48 h incubation was continued. After 48 h, planktonic bacteria were aspirated and removed, and each well was washed gently with sterile saline 0.9%, and the attached biofilm was kept [39].

### 4.4. In Vitro Photosensitization of S. mutans Biofilms for Optimization of the Dosimetry

The following parameters were investigated during the in vitro photosensitization: (1) TBO concentrations at three levels: 50, 100, and 150 μg/mL and (2) energy density at three levels: 36, 108, and 180 (Watts × second)/cm^2^ (Figure 1A). After determining the most effective concentration and energy density, the incubation time (pre-irradiation time) was investigated at three levels 1, 3, and 5 min considering only one concentration, 100 μg/mL.

Briefly, the biofilms were treated with the addition of 50 μL of 0.9% saline only (control) or TBO solution (photosensitizer) at prescreened concentrations of 50, 100, 150 μg/mL followed by the selected LED irradiation doses (aPDT treatment). Next, 50 µL of the photosensitizer solution was added to wells in which photosensitizer was tested alone to investigate their effect against the biofilm independently. Wells that were exposed to light without photosensitizer also served as control. Next, the experiment sets were repeated to investigate the preselected incubation times (1, 3, and 5 min prior to irradiation).

### 4.5. CFU Counting Assay

After treatment, the biofilms were removed and resuspended with 0.9% saline solution. Subsequently, the suspension was serially diluted (1:10, 1:100, 1:1000, 1:10,000, and 1:100,000) with 0.9% saline solution. Samples were plated in triplicate on B.H.I. agar and incubated for 48 h at 37 °C in a 5% CO_2_ incubator. Colonies of *S. mutans* were counted using a colony counter.

### 4.6. Live/Dead Staining of Biofilms

Random samples of 2-day biofilms treated via aPDT (100 μg/mL) and respective controls were washed with 0.9% saline and then stained with the BacLight live/dead kit (Molecular Probes, Eugene, OR, USA). A mixture of 2.5 μM SYTO 9 and 2.5 μM propidium iodide at a ratio of 1:1 was used to stain the wells for 10 min [40]. The green fluorescence of SYTO9 indicates the presence of viable *S. mutans*. The compromised *S. mutans* biofilms were indicated by red fluorescence. The images were taken using an inverted epifluorescence microscope (Eclipse TE2000-S, Nikon, Melville, NY, USA).

### 4.7. Statistical Analysis

Shapiro–Wilk test was used to evaluate the data normality and distribution. Then, two-way ANOVA and Tukey’s post hoc test were used to analyze the effect of TBO concentration and energy density on biofilm inhibition. For the cytotoxicity and the effect of the incubation time, one-way ANOVA and Tukey’s post hoc test were used. All tests were conducted using the statistical software package Sigma Plot 12.0 (S.Y.S.T.A.T., Chicago, IL, USA), and the statistical significance was set at *p* < 0.05.

## 5. Conclusions

Collectively, our data support the selection of primary determinants on the inactivation of *S. mutans* biofilms. Increasing the concentration of TBO and light energy dose was associated with increased biofilm inhibition. Increasing the incubation time was not associated with an increased antibacterial effect. The highest amount of biofilm inhibition with acceptable biocompatibility was achieved using 100 μg/mL of TBO activated by 180 J/cm^2^ energy dose. Future studies may consider investigating strategies to improve aPDT performance without manipulating the determinants related to dosimetry.
